# SOCS, SPRED, and NR4a: Negative regulators of cytokine signaling and transcription in immune tolerance

**DOI:** 10.2183/pjab.97.016

**Published:** 2021-06-11

**Authors:** Akihiko YOSHIMURA, Daisuke AKI, Minako ITO

**Affiliations:** *1 Department of Microbiology and Immunology, Keio University School of Medicine, Tokyo, Japan.; *2 Medical Institute of Bioregulation, Kyushu University, Fukuoka, Japan.

**Keywords:** immune tolerance, cytokine signal, regulatory T cells, autoimmunity, anergy

## Abstract

Cytokines are important intercellular communication tools for immunity. Most cytokines utilize the JAK-STAT and Ras-ERK pathways to promote gene transcription and proliferation; however, this signaling is tightly regulated. The suppressor of cytokine signaling (SOCS) family and SPRED family are a representative negative regulators of the JAK-STAT pathway and the Ras-ERK pathway, respectively. The SOCS family regulates the differentiation and function of CD4^+^ T cells, CD8^+^ T cells, and regulatory T cells, and is involved in immune tolerance, anergy, and exhaustion. SPRED family proteins have been shown to inactivate Ras by recruiting the Ras-GTPase neurofibromatosis type 1 (NF1) protein. Human genetic analysis has shown that SOCS family members are strongly associated with autoimmune diseases, allergies, and tumorigenesis, and SPRED1 is involved in NF1-like syndromes and tumors. We also identified the NR4a family of nuclear receptors as a key transcription factor for immune tolerance that suppresses cytokine expression and induces various immuno-regulatory molecules including SOCS1.

## 1 Introduction

Cytokines in the immune system are soluble molecules that mediate communication among immune cells or between immune cells and non-immune cells, often acting locally in a paracrine manner but sometimes acting systemically. Cytokines with antiviral activity are called interferons (IFNs), and factors that are involved in the proliferation and differentiation of hematopoietic cells are called hematopoietic factors, including erythropoietin (EPO) and granulocyte-colony stimulating factor (G-CSF). Many of these receptors belong to the structurally similar “cytokine receptor superfamily”.

The importance of cytokines in the immune response is clear. Inflammatory cytokines such as tumor necrosis factor (TNF)-α, interleukin (IL)-6, IL-12, and IL-23 are mainly produced by macrophages and dendritic cells to promote inflammation and activate the acquired immune system.^1)^ T cells of the acquired immune system include CD4^+^ helper T (Th) cells and CD8^+^ cytotoxic T cells (CTLs). Th cells are called the “commanders of immunity”; they release various T-cell cytokines, IFN-γ, IL-4, and IL-17, that activate B cells, CTLs, and cells of the innate immune system. Th cells that promote immunity are called effector T cells, and are currently classified into four main subsets: Th1, Th2, Th17, and T follicular helper (Tfh).^2)^ In addition, Th cell subsets contain regulatory T cells (Tregs), which suppress the immune system and produce suppressive cytokines such as IL-10 and transforming growth factor (TGF)-β.^3)^ Tregs are central to “immune tolerance”, a mechanism that controls excessive immune responses to “self”.^4)^

**Figure 1.**
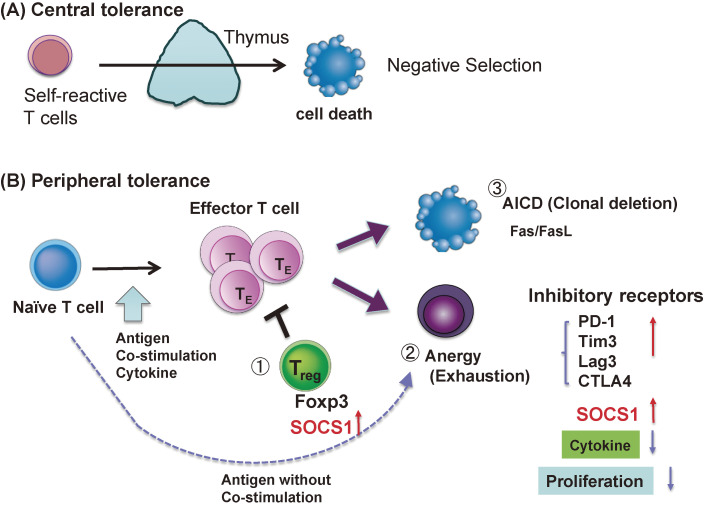
Mechanism of immune tolerance; central immune tolerance (A) and peripheral immune tolerance (B). Central immune tolerance involves “negative selection” in the thymus. Peripheral immune tolerance is mediated by regulatory T cells (Treg) (B①), anergy (or exhaustion) (B②), and AICD (clonal deletion) (B③).

Normally, inflammatory cytokines and T cells play a pivotal role in protective immunity against pathogens and tumors. T cells are also involved in the immune tolerance that suppresses excessive immune responses and inhibits reactions against self. Disruption of these immune tolerance mechanisms leads to autoimmune and allergic diseases. Without these mechanisms, individuals could not survive because they cannot intake food due to strong food allergies, and the human species might disappear because fetuses, half non-self, would be miscarried as a result of immune reactions. The mechanisms of immune tolerance are classified into two categories: central immune tolerance and peripheral immune tolerance (Fig. 1A,B). Central immune tolerance is the removal of self-reactive T cells by negative selection in the thymus. There are three major mechanisms of peripheral immune tolerance: Tregs, anergy, and clonal deletion (Fig. 1B).

Tregs are characterized by the expression of the transcription factor Forkhead box P3 (Foxp3), a master transcription factor of Tregs (Fig. 1B①). Tregs suppress excessive immune responses to a variety of antigens, including autoantigens, antigens from symbiotic bacteria, and exogenous antigens.^3)^ Tregs respond to T cell receptor (TCR) stimulation and IL-2, but are unresponsive to external cytokines and do not produce pro-inflammatory cytokines of their own.

Anergy is a state of nonresponsiveness that occurs when T cells are stimulated by antigens without a CD28 co-stimulatory signal. T cells in anergic state are no longer responsive to antigens or cytokines (Fig. 1B②). A similar state of dysfunction is called “exhaustion”, which occurs mostly in CD8^+^T cells chronically exposed to antigens and inflammatory signals in chronic infections or tumors. Both anergy and exhaustion are thought to be caused by the simultaneous expression of multiple inhibitory receptors, such as PD-1, Tim3, TIGIT, CTLA4, and Lag3, which carry a tyrosine phosphatase-recruiting motif (ITIM) as well as signal repressor molecules, such as suppressor of cytokine signaling (SOCS) and phosphatases, which render the cells unresponsive to stimuli.^5)^

Clonal deletion is a process in which T cells express Fas and Fas ligand (FasL) upon strong stimulation, leading to apoptosis (Fig. 1B③).^6)^ Thus, this process is called activation-induced cell death (AICD). Hyperactivation of T cells often causes clonal deletion, and cytokines such as IFN-γ play important roles in the induction of Fas and FasL.^7)^

Thus, the regulation of cytokine signaling that induces anergy, clonal deletion, and Tregs is essential for immune tolerance.

## 2 Signal transduction of cytokine receptors

**Figure 2.**
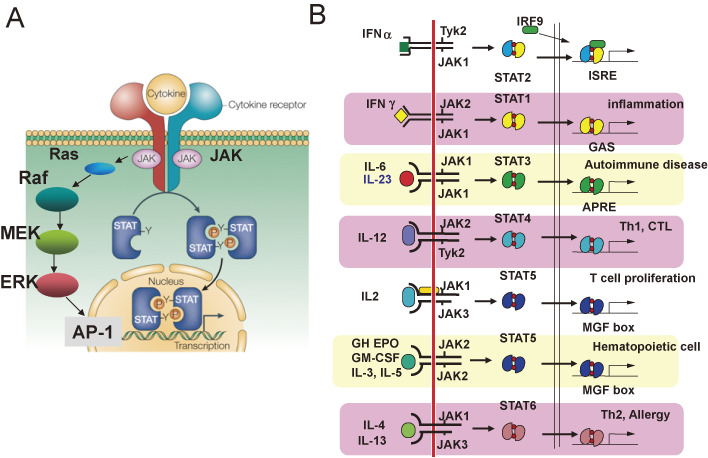
Signal transduction pathways of cytokine receptors (A) and the JAK-STAT pathway (B). (A) JAKs non-covalently associate with cytokine receptors, and are activated by cytokine binding to the receptors. The major downstream pathways of JAKs are STATs and Ras-ERK. Activated STATs are translocated to the nucleus from the cytoplasm and work as transcription factors. The Ras-ERK pathway activated various transcription factors, including AP-1. (B) There are four types of JAKs and six types of STATs, and their combination depends on the cytokine receptors.

The receptors for most cytokines activate the JAK-STAT pathway (Fig. 2A); receptors are included for immunoregulatory factors such as ILs and IFNs, hematopoietic factors such as G-CSF and EPO, and other endocrine cytokines such as Growth Hormone and Leptin.

### 2.1 Janus kinases.

Before the discovery of Janus kinases (JAKs), it was believed that Src-type tyrosine kinases were involved in cytokine receptor signaling. We reported that the EPO receptor is associated with the 130-kilodalton tyrosine phosphoprotein, pp130.^8)^ Subsequently, the pp130 associated with the EPO receptor was shown to be JAK2,^9)^ and it is now known that four JAK-type tyrosine kinases associate non-covalently with cytokine receptors and are activated by receptor oligomerization induced by cytokine binding.^10)^

There are four types of JAK-type tyrosine kinases, JAK1, 2, 3, and Tyk2, and each is associated with specific cytokine receptors. For example, JAK1 and Tyk2 bind to IFN-α/β receptors, JAK1 and JAK2 bind to IFN-γ receptors, JAK2 binds to IL-3 receptor β chain and EPO receptor, and JAK3 binds to IL-2 receptor γ chain (Fig. 2B).^11)^

When cytokines bind to their receptors, JAKs are activated through phosphorylation of the “kinase activation loop”, which undergoes a conformational change that allows substrates access to the enzyme catalytic pocket. Activated JAK then phosphorylates tyrosine residues on the receptors, which recruit intracellular signaling molecules that contain modules such as the SH2 domain and the phosphotyrosine-binding (PTB) domain that recognize phosphotyrosine motifs. These molecules bind to the phosphorylated receptor and are then phosphorylated by JAK and activated to transmit information further downstream. For example, in the case of EPO receptors, STAT5 is recruited to phosphorylated tyrosine (pY) 343, and pY401, phosphoinositide 3-kinase (PI3K) binds to pY429, and CIS (discussed below) and SH2-containing protein tyrosine phosphatase-1 (SHP-1) bind to pY429, and SHP-2 binds to pY401. SHP-1 negatively regulates signaling by dephosphorylating JAK2, whereas SHP-2 activates the Grb2-Ras-ERK pathway that promotes cell proliferation.^12,13)^

### 2.2 Signal transducers and activator of transcription.

Signal transducers and activator of transcription (STAT) is a characteristic protein with an SH2 domain and C-terminal tyrosine residue that is phosphorylated by JAKs (Fig. 2A). There are five classes of STAT family molecules (STAT5 consists of STAT5a and STAT5b) that are activated by specific cytokines (Fig. 2B). STAT1, mainly activated by IFN-γ, induces molecules involved in the promotion of immune reactions (Fc receptors, co-stimulatory molecules, MHC molecules, and cytotoxic molecules) and antiviral molecules. STAT3 is essential for the signaling of IL-6, leukemia inhibitory factor (LIF), oncostatin M (OSM), Leptin, and IL-10. STAT3 induces acute-phase proteins in response to IL-6 in the liver. In T cells, STAT3 is involved in the induction of RORγt, which is essential for Th17 cell differentiation. STAT4 is activated by IL-12 and induces Th1 cell differentiation. STAT5 induces casein and other milk proteins in the mammary glands. In T cells, the IL-2R α chain (CD25) is also a target gene of STAT5, and STAT5 is involved in the proliferation and survival of many hematopoietic and immune cells, including Tregs. STAT6 is activated by IL-4-related cytokines and plays important roles in Th2 responses.

### 2.3 Ras pathway.

Another important downstream pathway of cytokines is the Ras pathway (Fig. 2A). Activated Ras induces the activation of downstream extracellular signal-regulated kinase (ERK) and PI3K. The ERK pathway has been shown to be essential for the proliferation of T cells *in vitro*.^14)^ It also plays an important role in G-CSF-induced neutrophil production, and STAT3 negatively regulates neutrophil proliferation by inducing SOCS3 (discussed below).^15)^ The Ras-ERK pathway is thought to be essential for the proliferation of mast cells and eosinophils by IL-3 and IL-5, respectively.^16,17)^

## 3 Cytokine-inducible SH2 protein/suppressor of cytokine signaling family

The negative regulation of cytokine signaling in immunity is essential for maintaining immune-homeostasis and tolerance, suppressing excess immune responses, inducing anergy/exhaustion, and maintaining Tregs. Several mechanisms are known to regulate tyrosine kinase signaling, including SHP-1 tyrosine phosphatase, and the degradation of phosphotyrosine-based signaling molecules by c-Cbl.^18)^ However, these mechanisms are not specific to cytokine receptors. We discovered a new family of SH2 domain-containing proteins, the cytokine-inducible SH2 protein (CIS)/SOCS family, which are more specific to the JAK-STAT pathway of cytokine receptors. CIS was the first gene discovered in this family that was cloned as a cytokine-inducible gene.^19)^ The next discovered gene was SOCS1 (= JAB, SSI-1), which was reported simultaneously by three independent groups.^20–22)^ We isolated SOCS1 (also known as the JAK-binding protein, JAB) as a factor that binds to JAK and inhibits its kinase activity using a yeast two-hybrid system.^20)^ A database search revealed that there are currently eight members of this family.^23)^

**Figure 3.**
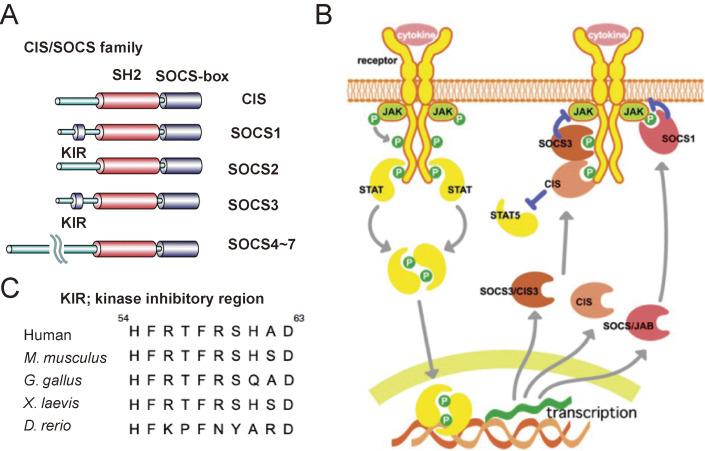
The CIS/SOCS family. (A) Basic structures of the CIS/SOCS family proteins. (B) CIS1, SOCS1, and SOCS3 are induced by STATs. CIS inhibits STAT5 activation by binding to the receptor, SOCS1 directly binds to JAK, and SOCS3 binds to both the gp130-related receptors and JAK. (C) Amino acid sequences of kinase inhibitory region (KIR) of SOCS1 of various species. The figures are modified from Ref. 58.

Among the CIS/SOCS family, genes with high specificity for cytokinesis are CIS (gene name *CISH* in humans), SOCS1, SOCS2, and SOCS3. In this family, the SH2 domain and the C-terminal SOCS-box are conserved (Fig. 3A). The role of SOCS-box will be described later. Because most SOCS family proteins are rapidly induced by cytokines, they are a major negative feedback regulator of cytokine signaling (Fig. 3B).

### 3.1 CIS and SOCS2.

CIS is transcriptionally induced by STAT5 and STAT6, which are activated by EPO, IL-2, IL-3, and IL-4, and binds to tyrosine-phosphorylated receptors mainly through the SH2 domain and inhibits STAT activation through the physical blocking of STAT recruitment to the phosphotyrosine residues of the receptor and degrades the receptors via SOCS-box-mediated ubiquitination (Fig. 3B).^24–26)^ CIS-deficient mice spontaneously develop asthma-like symptoms, and T cells have been shown to have increased sensitivity to IL-4.^27)^ Single nucleotide polymorphisms in the human *CISH* gene correlate with susceptibility to a number of infectious diseases, including tuberculosis and malaria,^28)^ and *Cis*^−/−^ mice also show resistance to tuberculosis infection.^29)^ CIS is also important in the regulation of the IL-15 sensitivity of natural killer (NK) cells,^30)^ and *CISH*^−/−^ NK cells derived from induced pluripotent stem cells have strong anti-tumor activity, which may be applicable to NK cell therapy for cancer.^31)^

SOCS2 associates with Growth Hormone receptors and regulates their signaling. Therefore, SOCS2-deficient mice exhibit giantis.^32)^ SOCS2 in dendritic cells also negatively regulates T cell activation in human cancer patients.^33)^

### 3.2 SOCS1- and SOCS3-deficient mice.

SOCS1 is highly expressed in thymic T cells as well as activated T cells.^34)^ SOCS1-deficient mice die of severe systemic inflammation, including fulminant hepatitis, by 3 weeks of age.^34)^ These phenotypes are ameliorated by treatment with anti-IFN-γ antibodies or by cross-fertilization with IFN-γ^−/−^ mice, and *Socs1* expression is strongly induced by IFN-γ, suggesting that SOCS1 is a potent negative regulator of IFNγ.^35)^ SOCS1 is also involved in the regulation of Toll-like receptor signaling in macrophages.^36,37)^ Tissue-specific deletion of the *Socs1* gene revealed that SOCS1 is fundamentally important for anti-inflammation, homeostasis, and immunological tolerance.^1,38–40)^ *Socs1*-deficient mice spontaneously developed intestinal tumors, and DNA methylation at the promoter site of the *SOCS1* gene is frequently found in human hepatocarcinoma, suggesting a relationship between reduced SOCS1 expression and inflammation-mediated tumor development.^40,41)^

SOCS3-deficient mice are prenatally lethal due to placental abnormalities caused by abnormal LIF signaling.^42)^ In addition, analyses of organ-specific knockout or transgenic mice have shown that SOCS3 plays an essential role in maintaining homeostasis in various tissues, such as the heart, brain, liver, fat, and joints.^43–46)^ Most phenotypes are due to the dysregulated signaling of cytokines that activate gp130-related receptors, such as cardiotrophin-1, Leptin, and IL-6. In addition, Th cell differentiation to Th17, which is promoted by IL-6, IL-23, and IL-27, is enhanced by SOCS3 deficiency.^47,48)^ In tumors, SOCS3 downregulation may contribute to hepatocarcinogenesis because STAT3 promotes hepatocarcinogenesis.^49)^

### 3.3 SOCS1 and SOCS3: molecular mechanisms of inhibition.

The SH2 domain of SOCS1 binds directly to the phosphorylated kinase activation loop of JAK, and the SH2 domain of SOCS3 binds with high affinity to the Y759 tyrosine residue of gp130 (Fig. 3B).^38,50,51)^ As suppression mechanisms, SOCS1 and SOCS3 have a kinase inhibitory peptide (kinase inhibitory region [KIR]) that directly suppresses the kinase activity of JAK in addition to the SOCS-box (Fig. 3A).^51)^ KIR is highly conserved among various species (Fig. 3C).

**Figure 4.**
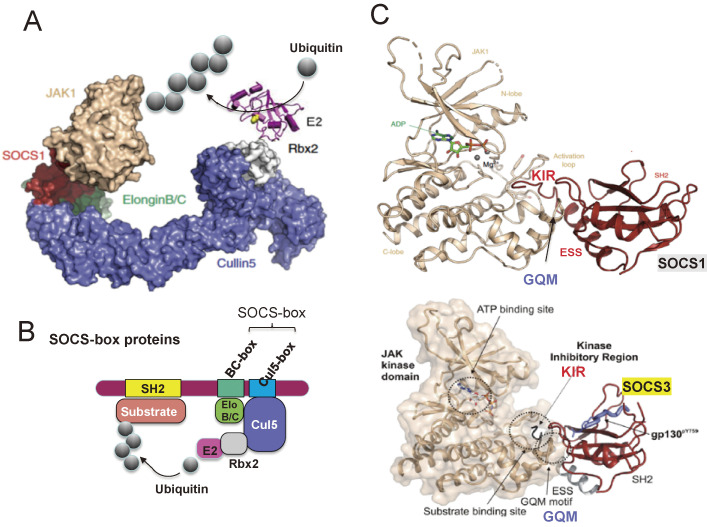
Molecular mechanisms of suppression by the SOCS-box and KIR. (A) X-ray crystallography of the complex of JAK, SOCS, and ubiquitin transfer system molecules. (B) Schematic model of SOCS-box-mediated ubiquitination of target molecules in the SH2 domain. The SOCS-box consists of BC-box and Cul5-box and recruits Elongin B/C heterodimers and Cullin 5 (Cul5). Cullin 5 also binds Rbx2, which recruits ubiquitin transferase E2. (C) X-ray crystallography of the complex of JAK and SOCS1 (upper) or SOCS3 (lower). There are no phosphorylated peptides in the SOCS1 SH2 domain in this structure, but the SOCS3 SH2 domain contains phosphopeptide of gp130. KIR; kinase inhibitory region, ESS; extended SH2 domain, GQM; GQM-motif of JAK. Structural figures are modified from Refs. 55–57.

The SOCS-box is found not only in the CIS/SOCS family but also in many other proteins including von Hippel-Lindau tumor suppressor protein, which are E3 ubiquitin ligases. The SOCS-box recruits ubiquitin transferases (Elongin B/C, Rbx1 and E2 enzyme complex) and promotes the ubiquitin-dependent proteasomal degradation of molecules, including receptors and JAKs that associate with the SH2 domain (see Fig. 4A,B).^52,53)^

We proposed an inhibitory mechanism in which the KIR acts as a pseudosubstrate and inhibits the binding of JAK substrates (Fig. 4C).^51,54)^ The noncanonical surface of the SH2 domain, that opposes the phosphopeptide-binding surface, and the N-terminal extended SH2 domain (ESS) of SOCS1 and SOCS3 bind to the GQM motif of JAK.^55–57)^ KIRs act as a pseudosubstrate and inhibits the binding of JAK substrates (Fig. 4C). Therefore, SOCS1 and SOCS3 do not inhibit the kinase activity of JAK3 because JAK3 does not have a GQM motif. The mechanism of inhibition determined by the crystal structure is almost identical to that predicted by our biochemical analysis.^1,51,58)^

An important function of SOCS3 is to determine the pro-inflammatory and anti-inflammatory effects of IL-6 and IL-10.^59)^ In macrophages, SOCS3 is induced by IL-6, and then associates with gp130, the IL-6 receptor, resulting in the transient activation of STAT3. In contrast, although SOCS3 is also induced by IL-10, it does not bind to the IL-10 receptor; therefore, STAT3 activation is strong and sustained. Hyperactivation of STAT3 suppresses NF-κB, which is activated by Toll-like receptor signaling, thus limiting the induction of inflammatory cytokines such as TNFα and IL-12. In macrophages expressing mutant gp130, which cannot associate with SOCS3, IL-6 behaves like IL-10, an anti-inflammatory cytokine. This model has been proven to be correct in a number of follow-up studies.^60,61)^

## 4 Sprouty-related protein with the EVH-1 domain (SPRED): Negative regulator of the Ras-ERK pathway

### 4.1 SPRED and RASopathy.

**Figure 5.**
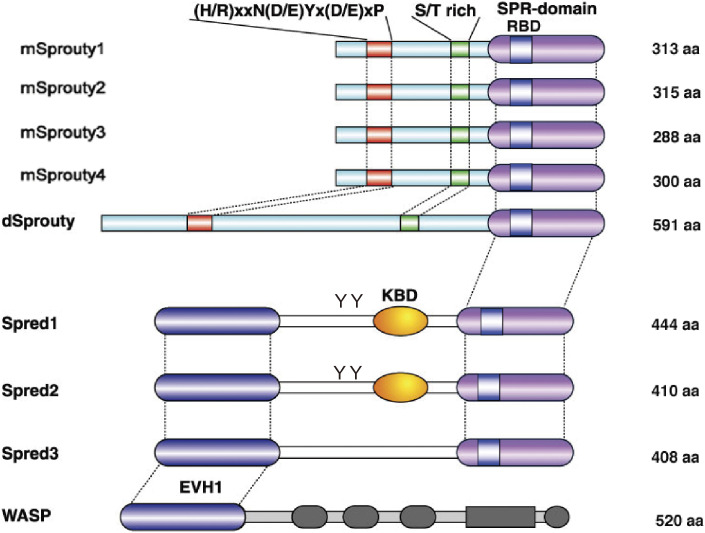
Structure of the mammalian Sprouty/Spred family proteins. dSprouty is the homologue of *Drosophila* Sprouty. Conserved N-terminal tyrosine motifs of Sproutys and other domains are illustrated. S/T rich; serine/threonine rich region, SPR; Sprouty-related domain, RBD; Raf-binding domain, KBD: c-kit binding domain, EVH1; Ena/VASP homology 1. YY in Spred1 and Spred2 indicates phosphorylated tyrosine residues. WASP; Wiskott-Aldrich syndrome protein. WASP contains a typical EVH1 domain. The figure is modified from Ref. 63.

There are a number of negative regulators of the Ras-ERK pathway, including GTPase-activating protein (GAP), which inactivates Ras and mitogen-activated protein kinase (MAPK) phosphatase (MKP), a phosphatase of ERK. Sprouty and Sprouty-related protein with the EVH-1 domain (SPRED) were newly discovered as negative regulators of the ERK pathway.^62,63)^ In mammals, there are four members of the Sprouty subfamily and three members in the SPRED subfamily (Fig. 5).^64)^ *In vitro*, mammalian Sprouty has been shown to regulate the phospholipase C (PLC)-mediated ERK pathway by growth factors,^65–67)^ whereas SPRED mainly regulates the Ras-dependent ERK pathway.^16,62)^

*Spred1*-deficient mice show hyperproliferation of myeloid cells, including neutrophils, eosinophils, mast cells, and type 2 innate lymphoid cells, due to the increased activation of the hematopoietic cytokines-dependent ERK pathway.^16,17,68,69)^ Murine Spred1 and Spred2 are important for the regulation and homeostasis of hematopoietic stem cells^70,71)^ as well as embryonic lymphangiogenesis.^72)^ *Spred1*-deficient mice also exhibit facial abnormalities, decreased learning and memory, and reduced synaptic plasticity.^69,73)^ All *in vitro* and *in vivo* data obtained from cultured cells, primary cells, and gene-targeted mice indicated that SPREDs are negative regulators of the Ras pathway.

**Figure 6.**
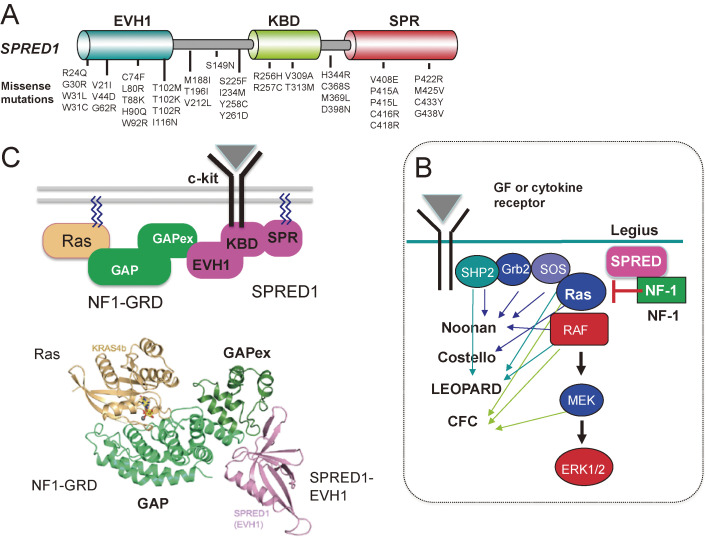
SPRED functions and RASopathy. (A) Schematic structure of SPRED1 and major mutations found in Legius syndrome. (B) Detailed signaling pathways of the Ras-ERK pathway from the cytokine or growth factor (GF) receptors, and related RASopathy syndromes. Mutations in the components of this pathway are linked to syndromes, including: Cardio-Facio-Cutaneous (CFC), Costello, Legius, Neurofibromatosis type 1 (NF1), Noonan, and LEOPARD syndromes. These syndromes share many clinical features such as distinct facial features, developmental delays, cardiac defects, growth delays, neurologic issues, and gastrointestinal difficulties. Not all components are shown in this figure. Please see Ref. 78 for details. (C) Model for the suppression of Ras activation by NF1 and SPRED1 complex. The SPRED1-EVH1 domain binds directly to the extended GAPex domain of NF1-GRD. SPRED1-KBD interacts with c-kit. SPR is palmitoylated and thus anchored in the membrane. The lower structure is the complex formed by Ras (brown), NF1-GRD (green), and SPRED1-EVH1 (pink) (modified from Refs. 80 and 86).

This hypothesis has been proven by the discovery of human disease-carrying SPRED1 mutations (Fig. 6A). Germline loss-of-function mutations of *SPRED1* were found in neurofibromatosis-1 (NF1)-like disease.^74,75)^ This disease is caused by the haplo-insufficiency of *SPRED1*, which is dominantly inherited, and causes café au lait macules, axillary freckling, macrocephaly, learning disabilities, and leukemia at low rates,^76)^ and is now called Legius syndrome.^77)^ The *NF1* gene encodes neurofibromin, a GAP that deactivates Ras. Legius syndrome and NF1 are part of a comprehensive syndrome called RASopathy (Fig. 6B), which is caused by germline loss-of-function and gain-of-function mutations in genes encoding protein components of the Ras/MAPK pathway.^78)^ These mutations result in excessive activation of the Ras-ERK pathway during the embryonic developmental stage. There are many phenotypic features that overlap among these syndromes, including characteristic facial features, cardiac defects, skin abnormalities, delayed neurocognition, and predisposition to malignancy.

SPREDs are also implicated in tumorigenesis.^79,80)^ Mutations in SPRED1 have been shown to be a tumor suppressor as they are found at a high frequency of >30% in KIT-driven mucosal melanoma.^81)^ Furthermore, homozygous deletions of SPRED1 contribute to resistance to MAPK-targeted therapy in melanoma patients.^82)^

### 4.2 Mechanism of suppression of Ras by SPRED.

SPRED has an EVH-1 domain at the N-terminus, c-kit binding domain (KBD) in the center, and a cysteine-rich (SPR) domain at the C-terminus that is homologous to Sprouty (Fig. 5). SPRED is anchored to the plasma membrane by palmitic oxidation of the C-terminal domain,^83)^ and it inhibits Ras activation by recruiting the NF1-GAP domain (GRD) via the N-terminal EVH1 domain (Fig. 6C).^84,85)^ We have identified amino acid residues that are important for the EVH1 domain to recognize GRD, based on the mutations found in human NF1 and Legius syndrome (see below).^84)^ The crystal structure of the complex of GRD, Ras, and the SPRED1-EVH1 domain revealed that the NF1-GRD has a unique domain for binding to the EVH1 domain of SPRED1 (Fig. 6C, lower) that is opposite to the Ras-binding site.^86)^ The GRD mutations found in human NF-1 are scattered among sites critical for the binding of GRD to SPRED1-EVH1.^84)^

## 5 SOCS1 and immune tolerance

### 5.1 SOCS1 mutations in human lymphoma.

Deletion mutations and functionally defective missense mutations of the *SOCS1* gene have been reported in many lymphomas, including Burkitt lymphoma and Hodgkin’s lymphoma.^87–89)^ SOCS1 deficiency is thought to result in the strong activation of JAK, which causes cell proliferation. These reports indicate that *SOCS1*, and probably *SOCS3*, are tumor suppressor genes in humans.

### 5.2 Human SOCS1 mutations and autoimmune diseases.

Genome-wide association studies (GWAS) have shown that SOCS1 single nucleotide polymorphisms (SNPs) are found in a variety of immune diseases, including primary biliary cirrhosis, multiple sclerosis, Crohn’s disease, and celiac disease, strongly suggesting the role of SOCS1 in immune regulation and human immunological diseases.^90)^ Although the phenotypes of SOCS1-deficient mice strongly indicate the important role of SOCS1 in immune tolerance, germline mutations of the *SOCS1* gene in humans have only recently been reported.

Germline mutations in the *SOCS1* gene were discovered in 2020. First, the whole genome sequencing of large sporadic (or non-familial) primary immunodeficiency disease patients in the United Kingdom discovered two heterozygous mutations.^91)^ Patients with p.Met161Alafs*46 and p.Tyr64* mutations showed decreased B cells, increased Th1 cells, and decreased Treg numbers. Patients with p.Met161Alafs*46 mutation also had lung and liver inflammation. T cells derived from patients with these *SOCS1* mutations showed reduced levels of SOCS1 protein and increased IFN-γ-induced phosphorylation of STAT1.

**Figure 7.**
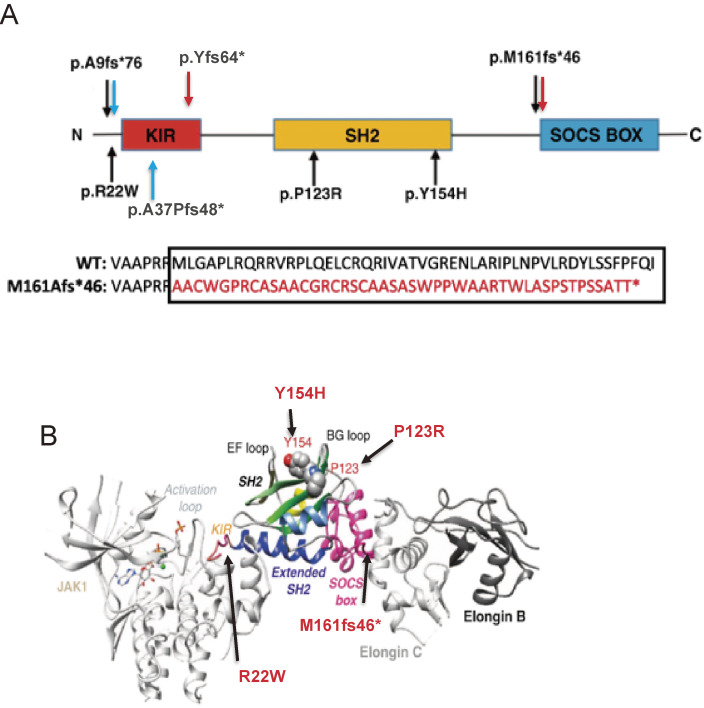
Mutations in *SOCS1*. (A) SOCS1 structure and mutations found in 5 families from France (black arrows),^92)^ two mutations found in the U.K. (red arrows),^91)^ and two mutation is the U.S. (blue arrows).^93)^ p.A9fs*76 and p.M161fs*46 were found in two cohorts. The SOCS1 M161Afs*46 mutant led to a predicted 46-residue neopeptide in the SOCS-box domain and disrupted the function of the SOCS-box. (B) Positions of mutations in the complex of JAK/SOCS1/Elongin B,C are shown. The two amino acids (P123 and Y154) are located in the phosphotyrosine peptide-binding groove of the SH2 domain; therefore, mutations P123R and Y154H probably impair the SH2 domain structure and/or function. Figures are modified from Ref. 92.

Next, heterozygous germline mutations in *SOCS1* were reported in 10 patients with early-onset autoimmune disease in five families from France (Fig. 7A,B).^92)^ The mutations are amino acid substitutions (c.368 C > G, p.P123R, c.64 C > T, p.R22W, c.460 T > C, p.Y154H) and deletions, all of which were considered loss-of-function (Fig. 7B). Symptoms are early-onset autoimmune disease; 60% of cases occur under 10 years of age and include immune thrombocytopenic purpura, psoriasis, celiac disease, systemic lupus erythematosus, thyroiditis, and hepatitis. Patients have higher levels of cytokines in the blood, similar to patients carrying gain-of-function mutations in STAT1 and STAT3. Some patients developed splenomegaly and Hodgkin’s lymphoma. Heterozygous lymphocytes with the mutation are more sensitive to cytokines such as IFN-γ, IL-2, and IL-4, and Treg functions are impaired.^92)^ Hyper STAT activation in these cells with *SOCS1* mutations was restored by the JAK1/JAK2 inhibitor ruxolitinib. T-cell activation was also increased in approximately 30% of asymptomatic carriers.

In addition, one sporadic case and one familial deletion of *SOCS1* have been reported in multisystem inflammatory syndrome in children (MIS-C) from the United States.^93)^ One patient developed a severe acute respiratory syndrome after SARS-CoV-2 infection, suggesting that *SOCS1*-deficiency may be related to the severity or prognostic symptoms of COVID-19. SOCS1 haploinsufficiency leads to early-onset autoimmune diseases associated with the cytokine hypersensitivity of immune cells. In other words, SOCS1 is an important gene for the maintenance of immune tolerance in humans.

### 5.3 SOCS1 and suppression of T cell activation.

SOCS1-heterozygous mice have increased Th1 differentiation and are susceptible to dextran sodium sulfate-induced colitis.^94)^ In order to elucidate the function of SOCS1 in T cells, conditional knockout (cKO) mice lacking SOCS1 specifically in T cells were generated. They developed autoimmune diseases such as dermatitis, splenomegaly, and hyperglobulinemia within a few months after birth.^95)^ CD4^+^ T cells in cKO mice are thought to be Th1 or Th2 dominant depending on their genetic background and environment. Peripheral T cells in cKO mice mostly showed activated memory types, and produced a much higher amount of IFN-γ than wild-type T cells whereas Th1 differentiation was rather suppressed due to Th1 predominance.^95)^

SOCS1 also plays an important role in suppressing activation in CD8^+^ T cells; the deletion of SOCS1 enhanced T cell responsiveness and resulted in strong anti-tumor immune activity.^96–98)^ Genome-wide CRISPR screening confirmed that SOCS1, Cbl-b, and other negative regulators are important for the restriction of human T cell proliferation and activation in response to TCR stimulation.^99)^ Of note, SOCS1 is considered to be a target of microRNA miR-155, and forced expression of miR-155 reduced the expression level of SOCS1 and enhanced its anti-tumor immunity.^100)^ Furthermore, miR-155 deficiency attenuated hepatic ischemia–reperfusion injury via *Socs1* upregulation.^101)^ These results suggest that SOCS1 acts as an immune checkpoint molecule in obliterating effector T cells and is involved in T cell anergy.

### 5.4 SOCS1 and Tregs.

SOCS1 also plays an important role in the regulation of Tregs.^102)^ SOCS1 is consistently highly expressed in Treg (see Fig. 1), and Treg-specific *Socs1*-deficient mice exhibit symptoms of inflammatory diseases, such as dermatitis, hepatitis, and splenomegaly.^103,104)^ *Socs1*-deficient Tregs lose Foxp3 expression and convert to Th1 or Th17-like cells, which produce IFN-γ and IL-17, respectively, possibly due to STAT1 and STAT3 hyperactivation.^104)^ It has been reported that Ubc13 regulates Treg effector cytokine signaling molecules, including SOCS1, and is involved in their suppressive activity.^105)^ Loss of Treg function has also been reported in human heterozygous SOCS1 mutation carriers.^92)^

The TGF-β/Smad pathway has been shown to be necessary for Foxp3 expression in peripheral naïve CD4^+^ T cells, which are called peripheral Tregs (pTregs).^2,106)^ The phenotype of Smad2/3-deficient Tregs is similar to that observed in SOCS1-deficient Tregs. This is probably because overactivation of STAT1 by SOCS1-deficiency suppresses the TGF-β/Smad pathway, therefore causing Foxp3 instability.^95)^

SOCS1 mRNA is a target of miR-155.^103,107)^ Upregulation of Foxp3 is associated with high miR-155 expression in Tregs, where *Socs1* expression is downregulated. The importance of *Socs1* as a target gene of miR-155 was shown by disrupting the miR-155 binding site in the *Socs1* 3′UTR, indicating that this axis is important for Tregs.^108)^ Conversely, miR-146a targets STAT1 and thereby regulates *Socs1* expression.^107)^

## 6 NR4a, a transcription factor responsible for T cell tolerance and exhaustion

As mentioned above, SOCS1 confers cytokine unresponsiveness in T cells and thus plays an important role in the anergic properties of effector T cells and cytokine insensitivity of Tregs. Anergy and exhaustion are dependent on the simultaneous expression of multiple inhibitory receptors, such as PD-1, Tim3, and LAG3, as well as signal repressor molecules, such as SOCS1.^5)^ Next, we have sought to identify the transcription factor that upregulates SOCS1 and inhibitory receptors and is required for T cell anergy and Treg maintenance. Although Tregs are essential for immune tolerance, little is known about the key transcription factor that induces Foxp3 in thymic CD4^+^ T cells.

### 6.1 NR4a factors are essential for Treg development and maintenance.

We found that NR4a factors are essential for the development of Tregs.^109,110)^ The NR4a family consists of NR4A1 (also called NUR77), NR4A2 (NURR1), and NR4A3 (NOR1), which are nuclear orphan receptor-type transcription factors. These three factors are thought to recognize the same DNA sequences because their DNA-binding domains are almost identical in structure. They are widely conserved from *C. elegans* to mammals and have diverse functions such as metabolic regulation, lifespan, and differentiation control. Similar to other nuclear receptors, NR4a can function as a monomer, homodimer, or heterodimer.

**Figure 8.**
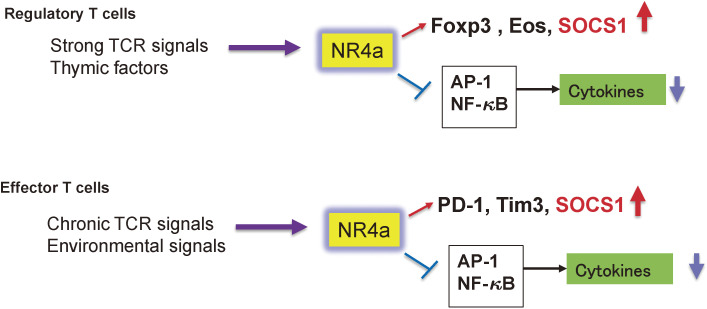
Role of NR4a in peripheral immune tolerance. NR4a is induced by strong TCR stimulation in Treg precursors in the thymus and is maintained at high levels after development. NR4a enhances the expression of Foxp3, Eos, and SOCS1 and inhibits the expression of inflammatory cytokines through blocking AP-1 and NF-κB. In effector T cells, NR4a induces the expression of inhibitory receptors, including PD-1, Tim3, and SOCS1, while suppressing cytokine expression by regulating NF-κB and AP-1.

NR4a mainly binds to the promoter region and activates the transcription of Foxp3, the master transcription factor of Tregs. All three NR4a factors have been shown to be induced by continuous TCR signals and are highly expressed in Tregs, especially in the thymus, because Tregs develop by strong TCR signals through the recognition of self-antigens (Fig. 8).^5)^ Forced expression of NR4a1, 2, or 3 in naïve T cells induces Foxp3 expression, whereas CD4^+^ T cells lacking NR4a1, 2, and 3 do not generate Tregs in the thymus.^109)^ Thus, NR4a is a transcription factor that is essential for the induction of Treg in the thymus. In addition, NR4a is required for Treg maintenance, and NR4a-deficient Tregs readily lose Foxp3 expression and transform into Th2 and Tfh cells.^111)^ NR4a promotes Foxp3 and Ikzf4 (Eos) expression and suppresses cytokine gene expression, including IL-4, IL-21, and IFN-γ (Fig. 8).

### 6.2 Regulation of T cell anergy and exhaustion by NR4a.

In effector T cells, NR4a has been shown to be essential for T cell anergy and exhaustion.^5)^ T cell exhaustion has been studied using various models, but in particular using an NFAT mutant. NFAT normally induces T cell activation in response to antigen stimulation, but without co-stimulation NFAT induces anergy or exhaustion. Thus, forced expression of an NFAT mutant lacking the AP-1 binding site induced T cell anergy/exhaustion.^112)^ Genome-wide ATAC-seq analysis was performed in activated T cells and exhausted T cells. Consensus binding sites for NFAT and NR4a family members were found in open regions of chromosomes unique to exhausted T cells, such as PD-1 and Tim3 (Fig. 8).^113)^ Overexpression of each NR4a factor resulted in a gene expression profile similar to that of exhausted T cells.^114)^ Conversely, T cells lacking the NR4a transcription factors showed a reduced PD-1^high+^Tim3^+^ exhausted fraction.^114)^ ATAC-seq analysis revealed that NR4a directly activates the genes involved in T cell inactivation such as PD-1 and Tim3, whereas it suppresses effector gene expression such as IFN-γ and TNFα by blocking AP-1 and NF-κB (Fig. 8). Similar results were found using a T cell anergy model obtained by stimulating T cells with an antigen but without co-stimulation^115)^ and Tregs (Fig. 8).^116)^ Thus, NR4a is a type of master regulator of anergy/exhaustion in T cells. Most notably, the forced expression of NR4a up-regulated *Socs1* expression, and the promoter region of the *Socs1* gene contains a binding site for NR4a (unpublished data).

## 7 Conclusion

As described above, for the past 30 years, we have been working to elucidate the molecular mechanisms of the regulation of cytokine signaling by the CIS/SOCS and SPRED families and their physiological and pathological significance. These studies have inspired many researchers. The molecular mechanism of suppression has been demonstrated at the atomic level by X-ray crystallography, and both SOCS1 and SPRED1 have been confirmed as human disease genes. We have shown that SOCS is necessary for immune tolerance, and we have further identified the NR4a family of transcription factors as the hub of immune tolerance.

The importance of negative regulators such as SOCS in immunity is recognized today as inducers of “immune tolerance” in effector T cells as well as in Tregs. In addition, NR4a family members have been cloned as factors required for T cell exhaustion and anergy and for the development and maintenance of Tregs. We have shown that SOCS1 is involved in tolerance by regulating cytokine signaling, and that NR4a induces tolerance by positively and negatively regulating effector and inhibitory genes transcription. We are fortunate to have discovered two gene families involved in immune tolerance, which is a key feature of immunity. It is easy to imagine that SOCS1 and NR4a act as immune checkpoints in tumor immunity. In mouse models, we have shown that the deletion of SOCS1 or NR4a in T cells regresses cancers due to strong tumor immunity.^96,117)^ We will continue our research in pursuit of the application of SOCS and NR4a to the treatment of autoimmune diseases and cancers in humans.

## References

[1] Yoshimura A. Mori H. Ohishi M. Aki D. Hanada T. (2003) Negative regulation of cytokine signaling influences inflammation. Curr. Opin. Immunol. 15, 704–708. 14630206 10.1016/j.coi.2003.09.004

[2] Yoshimura A. Muto G. (2011) TGF-β function in immune suppression. Curr. Top. Microbiol. Immunol. 350, 127–147. 20680806 10.1007/82_2010_87

[3] Kanamori M. Nakatsukasa H. Okada M. Lu Q. Yoshimura A. (2016) Induced regulatory T cells: Their development, stability, and applications. Trends Immunol. 37, 803–811. 27623114 10.1016/j.it.2016.08.012

[4] Ito M. Komai K. Nakamura T. Srirat T. Yoshimura A. (2019) Tissue regulatory T cells and neural repair. Int. Immunol. 31, 361–369. 30893423 10.1093/intimm/dxz031

[5] Ando M. Ito M. Srirat T. Kondo T. Yoshimura A. (2020) Memory T cell, exhaustion, and tumor immunity. Immun. Med. 43, 1–9. 10.1080/25785826.2019.1698261 31822213

[6] Green D.R. Droin N. Pinkoski M. (2003) Activation-induced cell death in T cells. Immunol. Rev. 193, 70–81. 12752672 10.1034/j.1600-065x.2003.00051.x

[7] De Rose V. Cappello P. Sorbello V. Ceccarini B. Gani F. Bosticardo M. (2004) IFN-γ inhibits the proliferation of allergen-activated T lymphocytes from atopic, asthmatic patients by inducing Fas/FasL-mediated apoptosis. J. Leukoc. Biol. 76, 423–432. 15123769 10.1189/jlb.0503247

[8] Yoshimura A. Lodish H.F. (1992) In vitro phosphorylation of the erythropoietin receptor and an associated protein, pp130. Mol. Cell. Biol. 12, 706–715. 1310150 10.1128/mcb.12.2.706-715.1992 PMC364272

[9] Witthuhn B.A. Quelle F.W. Silvennoinen O. Yi T. Tang B. Miura O. (1993) JAK2 associates with the erythropoietin receptor and is tyrosine phosphorylated and activated following stimulation with erythropoietin. Cell 74, 227–236. 8343951 10.1016/0092-8674(93)90414-l

[10] Schwartz D.M. Bonelli M. Gadina M. O’Shea J.J. (2016) Type I/II cytokines, JAKs, and new strategies for treating autoimmune diseases. Nat. Rev. Rheumatol. 12, 25–36. 26633291 10.1038/nrrheum.2015.167 PMC4688091

[11] O’Shea J.J. Gadina M. Schreiber R.D. (2002) Cytokine signaling in 2002: New surprises in the Jak/Stat pathway. Cell 109, S121–S131. 11983158 10.1016/s0092-8674(02)00701-8

[12] Debeljak N. Solár P. Sytkowski A.J. (2014) Erythropoietin and cancer: The unintended consequences of anemia correction. Front. Immunol. 5, 563. 25426117 10.3389/fimmu.2014.00563 PMC4227521

[13] Yoshimura A. Misawa H. (1998) Physiology and function of the erythropoietin receptor. Curr. Opin. Hematol. 5, 171–176. 9664155 10.1097/00062752-199805000-00004

[14] Sakamoto H. Kitamura T. Yoshimura A. (2000) Mitogen-activated protein kinase plays an essential role in the erythropoietin-dependent proliferation of CTLL-2 cells. J. Biol. Chem. 275, 35857–35862. 10960479 10.1074/jbc.M006317200

[15] Kimura A. Kinjyo I. Matsumura Y. Mori H. Mashima R. Harada M. (2004) SOCS3 is a physiological negative regulator for granulopoiesis and granulocyte colony-stimulating factor receptor signaling. J. Biol. Chem. 279, 6905–6910. 14699146 10.1074/jbc.C300496200

[16] Nonami A. Kato R. Taniguchi K. Yoshiga D. Taketomi T. Fukuyama S. (2004) Spred-1 negatively regulates interleukin-3-mediated ERK/mitogen-activated protein (MAP) kinase activation in hematopoietic cells. J. Biol. Chem. 279, 52543–52551. 15465815 10.1074/jbc.M405189200

[17] Inoue H. Kato R. Fukuyama S. Nonami A. Taniguchi K. Matsumoto K. (2005) Spred-1 negatively regulates allergen-induced airway eosinophilia and hyperresponsiveness. J. Exp. Med. 201, 73–82. 15630138 10.1084/jem.20040616 PMC2212755

[18] Yokouchi M. Kondo T. Sanjay A. Houghton A. Yoshimura A. Komiya S. (2001) Src-catalyzed phosphorylation of c-Cbl leads to the interdependent ubiquitination of both proteins. J. Biol. Chem. 276, 35185–35193. 11448952 10.1074/jbc.M102219200

[19] Yoshimura A. Ohkubo T. Kiguchi T. Jenkins N.A. Gilbert D.J. Copeland N.G. (1995) A novel cytokine-inducible gene CIS encodes an SH2-containing protein that binds to tyrosine-phosphorylated interleukin 3 and erythropoietin receptors. EMBO J. 14, 2816–2826. 7796808 10.1002/j.1460-2075.1995.tb07281.x PMC398400

[20] Endo T.A. Masuhara M. Yokouchi M. Suzuki R. Sakamoto H. Mitsui K. (1997) A new protein containing an SH2 domain that inhibits JAK kinases. Nature 387, 921–924. 9202126 10.1038/43213

[21] Naka T. Narazaki M. Hirata M. Matsumoto T. Minamoto S. Aono A. (1997) Structure and function of a new STAT-induced STAT inhibitor. Nature 387, 924–929. 9202127 10.1038/43219

[22] Starr R. Willson T.A. Viney E.M. Murray L.J. Rayner J.R. Jenkins B.J. (1997) A family of cytokine-inducible inhibitors of signalling. Nature 387, 917–921. 9202125 10.1038/43206

[23] Masuhara M. Sakamoto H. Matsumoto A. Suzuki R. Yasukawa H. Mitsui K. (1997) Cloning and characterization of novel CIS family genes. Biochem. Biophys. Res. Commun. 239, 439–446. 9344848 10.1006/bbrc.1997.7484

[24] Matsumoto A. Masuhara M. Mitsui K. Yokouchi M. Ohtsubo M. Misawa H. (1997) CIS, a cytokine inducible SH2 protein, is a target of the JAK-STAT5 pathway and modulates STAT5 activation. Blood 89, 3148–3154. 9129017

[25] Matsumoto A. Seki Y. Kubo M. Ohtsuka S. Suzuki A. Hayashi I. (1999) Suppression of STAT5 functions in liver, mammary glands, and T cells in cytokine-inducible SH2-containing protein 1 transgenic mice. Mol. Cell. Biol. 19, 6396–6407. 10454585 10.1128/mcb.19.9.6396 PMC84609

[26] Verdier F. Chrétien S. Muller O. Varlet P. Yoshimura A. Gisselbrecht S. (1998) Proteasomes regulate erythropoietin receptor and signal transducer and activator of transcription 5 (STAT5) activation. Possible involvement of the ubiquitinated Cis protein. J. Biol. Chem. 273, 28185–28190. 9774439 10.1074/jbc.273.43.28185

[27] Yang X.X.O. Zhang H.Y. Kim B.S. Niu X.Y. Peng J. Chen Y.H. (2013) The signaling suppressor CIS controls proallergic T cell development and allergic airway inflammation. Nat. Immunol. 14, 732–740. 23727894 10.1038/ni.2633 PMC4084713

[28] Khor C.C. Vannberg F.O. Chapman S.J. Guo H. Wong S.H. Walley A.J. (2010) CISH and susceptibility to infectious diseases. N. Engl. J. Med. 362, 2092–2101. 20484391 10.1056/NEJMoa0905606 PMC3646238

[29] Queval C.J. Song O.R. Carralot J.P. Saliou J.M. Bongiovanni A. Deloison G. (2017) *Mycobacterium tuberculosis* controls phagosomal acidification by targeting CISH-mediated signaling. Cell Rep. 20, 3188–3198. 28954234 10.1016/j.celrep.2017.08.101 PMC5637157

[30] Delconte R.B. Kolesnik T.B. Dagley L.F. Rautela J. Shi W. Putz E.M. (2016) CIS is a potent checkpoint in NK cell-mediated tumor immunity. Nat. Immunol. 17, 816–824. 27213690 10.1038/ni.3470

[31] Zhu H. Blum R.H. Bernareggi D. Ask E.H. Wu Z. Hoel H.J. (2020) Metabolic reprograming via deletion of CISH in human iPSC-derived NK cells promotes in vivo persistence and enhances anti-tumor activity. Cell Stem Cell 27, 224–237.e226. 32531207 10.1016/j.stem.2020.05.008 PMC7415618

[32] Greenhalgh C.J. Rico-Bautista E. Lorentzon M. Thaus A.L. Morgan P.O. Willson T.A. (2005) SOCS2 negatively regulates growth hormone action in vitro and in vivo. J. Clin. Invest. 115, 397–406. 15690087 10.1172/JCI22710 PMC546423

[33] Nirschl C.J. Suárez-Fariñas M. Izar B. Prakadan S. Dannenfelser R. Tirosh I. (2017) IFNγ-dependent tissue-immune homeostasis is co-opted in the tumor microenvironment. Cell 170, 127–141.e115. 28666115 10.1016/j.cell.2017.06.016 PMC5569303

[34] Marine J.C. Topham D.J. McKay C. Wang D. Parganas E. Stravopodis D. (1999) SOCS1 deficiency causes a lymphocyte-dependent perinatal lethality. Cell 98, 609–616. 10490100 10.1016/s0092-8674(00)80048-3

[35] Sakamoto H. Kinjyo I. Yoshimura A. (2000) The Janus kinase inhibitor, Jab/SOCS-1, is an interferon-γ inducible gene and determines the sensitivity to interferons. Leuk. Lymphoma 38, 49–58. 10811447 10.3109/10428190009060318

[36] Kinjyo I. Hanada T. Inagaki-Ohara K. Mori H. Aki D. Ohishi M. (2002) SOCS1/JAB is a negative regulator of LPS-induced macrophage activation. Immunity 17, 583–591. 12433365 10.1016/s1074-7613(02)00446-6

[37] Naka T. Fujimoto M. Tsutsui H. Yoshimura A. (2005) Negative regulation of cytokine and TLR signalings by SOCS and others. Adv. Immunol. 87, 61–122. 16102572 10.1016/S0065-2776(05)87003-8

[38] Yoshimura A. Naka T. Kubo M. (2007) SOCS proteins, cytokine signalling and immune regulation. Nat. Rev. Immunol. 7, 454–465. 17525754 10.1038/nri2093

[39] Hanada T. Kinjyo I. Inagaki-Ohara K. Yoshimura A. (2003) Negative regulation of cytokine signaling by CIS/SOCS family proteins and their roles in inflammatory diseases. Rev. Physiol. Biochem. Pharmacol. 149, 72–86. 12687406 10.1007/s10254-003-0015-z

[40] Hanada T. Kobayashi T. Chinen T. Saeki K. Takaki H. Koga K. (2006) IFNγ-dependent, spontaneous development of colorectal carcinomas in SOCS1-deficient mice. J. Exp. Med. 203, 1391–1397. 16717119 10.1084/jem.20060436 PMC2118311

[41] Yoshida T. Ogata H. Kamio M. Joo A. Shiraishi H. Tokunaga Y. (2004) SOCS1 is a suppressor of liver fibrosis and hepatitis-induced carcinogenesis. J. Exp. Med. 199, 1701–1707. 15197228 10.1084/jem.20031675 PMC2212816

[42] Takahashi Y. Carpino N. Cross J.C. Torres M. Parganas E. Ihle J.N. (2003) SOCS3: An essential regulator of LIF receptor signaling in trophoblast giant cell differentiation. EMBO J. 22, 372–384. 12554639 10.1093/emboj/cdg057 PMC140741

[43] Hamanaka I. Saito Y. Yasukawa H. Kishimoto I. Kuwahara K. Miyamoto Y. (2001) Induction of JAB/SOCS-1/SSI-1 and CIS3/SOCS-3/SSI-3 is involved in gp130 resistance in cardiovascular system in rat treated with cardiotrophin-1 in vivo. Circ. Res. 88, 727–732. 11304496 10.1161/hh0701.088512

[44] Shouda T. Yoshida T. Hanada T. Wakioka T. Oishi M. Miyoshi K. (2001) Induction of the cytokine signal regulator SOCS3/CIS3 as a therapeutic strategy for treating inflammatory arthritis. J. Clin. Invest. 108, 1781–1788. 11748261 10.1172/JCI13568 PMC209467

[45] Torisu T. Sato N. Yoshiga D. Kobayashi T. Yoshioka T. Mori H. (2007) The dual function of hepatic SOCS3 in insulin resistance in vivo. Genes Cells 12, 143–154. 17295835 10.1111/j.1365-2443.2007.01044.x

[46] Yasukawa H. Hoshijima M. Gu Y. Nakamura T. Pradervand S. Hanada T. (2001) Suppressor of cytokine signaling-3 is a biomechanical stress-inducible gene that suppresses gp130-mediated cardiac myocyte hypertrophy and survival pathways. J. Clin. Invest. 108, 1459–1467. 11714737 10.1172/JCI13939 PMC209425

[47] Stumhofer J.S. Laurence A. Wilson E.H. Huang E. Tato C.M. Johnson L.M. (2006) Interleukin 27 negatively regulates the development of interleukin 17-producing T helper cells during chronic inflammation of the central nervous system. Nat. Immunol. 7, 937–945. 16906166 10.1038/ni1376

[48] Taleb S. Romain M. Ramkhelawon B. Uyttenhove C. Pasterkamp G. Herbin O. (2009) Loss of SOCS3 expression in T cells reveals a regulatory role for interleukin-17 in atherosclerosis. J. Exp. Med. 206, 2067–2077. 19737863 10.1084/jem.20090545 PMC2757872

[49] Ogata H. Kobayashi T. Chinen T. Takaki H. Sanada T. Minoda Y. (2006) Deletion of the *SOCS3* gene in liver parenchymal cells promotes hepatitis–induced hepatocarcinogenesis. Gastroenterology 131, 179–193. 16831601 10.1053/j.gastro.2006.04.025

[50] Suzuki R. Sakamoto H. Yasukawa H. Masuhara M. Wakioka T. Sasaki A. (1998) CIS3 and JAB have different regulatory roles in interleukin-6 mediated differentiation and STAT3 activation in M1 leukemia cells. Oncogene 17, 2271–2278. 9811457 10.1038/sj.onc.1202143

[51] Yasukawa H. Misawa H. Sakamoto H. Masuhara M. Sasaki A. Wakioka T. (1999) The JAK-binding protein JAB inhibits Janus tyrosine kinase activity through binding in the activation loop. EMBO J. 18, 1309–1320. 10064597 10.1093/emboj/18.5.1309 PMC1171221

[52] Kamura T. Sato S. Haque D. Liu L. Kaelin W.G. Jr. Conaway R.C. (1998) The Elongin BC complex interacts with the conserved SOCS-box motif present in members of the SOCS, ras, WD-40 repeat, and ankyrin repeat families. Genes Dev. 12, 3872–3881. 9869640 10.1101/gad.12.24.3872 PMC317264

[53] Kamizono S. Hanada T. Yasukawa H. Minoguchi S. Kato R. Minoguchi M. (2001) The SOCS box of SOCS-1 accelerates ubiquitin-dependent proteolysis of TEL-JAK2. J. Biol. Chem. 276, 12530–12538. 11278610 10.1074/jbc.M010074200

[54] Sasaki A. Yasukawa H. Suzuki A. Kamizono S. Syoda T. Kinjyo I. (1999) Cytokine-inducible SH2 protein-3 (CIS3/SOCS3) inhibits Janus tyrosine kinase by binding through the N-terminal kinase inhibitory region as well as SH2 domain. Genes Cells 4, 339–351. 10421843 10.1046/j.1365-2443.1999.00263.x

[55] Babon J.J. Kershaw N.J. Murphy J.M. Varghese L.N. Laktyushin A. Young S.N. (2012) Suppression of cytokine signaling by SOCS3: Characterization of the mode of inhibition and the basis of its specificity. Immunity 36, 239–250. 22342841 10.1016/j.immuni.2011.12.015 PMC3299805

[56] Liau N.P.D. Laktyushin A. Lucet I.S. Murphy J.M. Yao S. Whitlock E. (2018) The molecular basis of JAK/STAT inhibition by SOCS1. Nat. Commun. 9, 1558. 29674694 10.1038/s41467-018-04013-1 PMC5908791

[57] Kershaw N.J. Murphy J.M. Liau N.P. Varghese L.N. Laktyushin A. Whitlock E.L. (2013) SOCS3 binds specific receptor-JAK complexes to control cytokine signaling by direct kinase inhibition. Nat. Struct. Mol. Biol. 20, 469–476. 23454976 10.1038/nsmb.2519 PMC3618588

[58] Yoshimura A. Nishinakamura H. Matsumura Y. Hanada T. (2005) Negative regulation of cytokine signaling and immune responses by SOCS proteins. Arthritis Res. Ther. 7, 100–110. 15899058 10.1186/ar1741 PMC1174965

[59] Yasukawa H. Ohishi M. Mori H. Murakami M. Chinen T. Aki D. (2003) IL-6 induces an anti-inflammatory response in the absence of SOCS3 in macrophages. Nat. Immunol. 4, 551–556. 12754507 10.1038/ni938

[60] El Kasmi K.C. Holst J. Coffre M. Mielke L. de Pauw A. Lhocine N. (2006) General nature of the STAT3-activated anti-inflammatory response. J. Immunol. 177, 7880–7888. 17114459 10.4049/jimmunol.177.11.7880

[61] Fowler K.D. Kuchroo V.K. Chakraborty A.K. (2012) A model for how signal duration can determine distinct outcomes of gene transcription programs. PLoS One 7, e33018. 22427931 10.1371/journal.pone.0033018 PMC3302786

[62] Wakioka T. Sasaki A. Kato R. Shouda T. Matsumoto A. Miyoshi K. (2001) Spred is a Sprouty-related suppressor of Ras signalling. Nature 412, 647–651. 11493923 10.1038/35088082

[63] Yoshimura A. (2009) Regulation of cytokine signaling by the SOCS and Spred family proteins. Keio J. Med. 58, 73–83. 19597303 10.2302/kjm.58.73

[64] Kato R. Nonami A. Taketomi T. Wakioka T. Kuroiwa A. Matsuda Y. (2003) Molecular cloning of mammalian Spred-3 which suppresses tyrosine kinase-mediated Erk activation. Biochem. Biophys. Res. Commun. 302, 767–772. 12646235 10.1016/s0006-291x(03)00259-6

[65] Sasaki A. Taketomi T. Kato R. Saeki K. Nonami A. Sasaki M. (2003) Mammalian Sprouty4 suppresses Ras-independent ERK activation by binding to Raf1. Nat. Cell Biol. 5, 427–432. 12717443 10.1038/ncb978

[66] Ayada T. Taniguchi K. Okamoto F. Kato R. Komune S. Takaesu G. (2009) Sprouty4 negatively regulates protein kinase C activation by inhibiting phosphatidylinositol 4,5-biphosphate hydrolysis. Oncogene 28, 1076–1088. 19137008 10.1038/onc.2008.464

[67] Taniguchi K. Ishizaki T. Ayada T. Sugiyama Y. Wakabayashi Y. Sekiya T. (2009) Sprouty4 deficiency potentiates Ras-independent angiogenic signals and tumor growth. Cancer Sci. 100, 1648–1654. 19493272 10.1111/j.1349-7006.2009.01214.x PMC11158288

[68] Suzuki M. Morita R. Hirata Y. Shichita T. Yoshimura A. (2015) Spred1, a suppressor of the Ras-ERK pathway, negatively regulates expansion and function of group 2 innate lymphoid cells. J. Immunol. 195, 1273–1281. 26116510 10.4049/jimmunol.1500531

[69] Ishizaki T. Tamiya T. Taniguchi K. Morita R. Kato R. Okamoto F. (2011) miR126 positively regulates mast cell proliferation and cytokine production through suppressing Spred1. Genes Cells 16, 803–814. 21668589 10.1111/j.1365-2443.2011.01529.x

[70] Tadokoro Y. Hoshii T. Yamazaki S. Eto K. Ema H. Kobayashi M. (2018) Spred1 safeguards hematopoietic homeostasis against diet-induced systemic stress. Cell Stem Cell 22, 713–725.e718. 29706577 10.1016/j.stem.2018.04.002

[71] Nobuhisa I. Kato R. Inoue H. Takizawa M. Okita K. Yoshimura A. (2004) Spred-2 suppresses aorta-gonad-mesonephros hematopoiesis by inhibiting MAP kinase activation. J. Exp. Med. 199, 737–742. 14981116 10.1084/jem.20030830 PMC2213301

[72] Taniguchi K. Kohno R. Ayada T. Kato R. Ichiyama K. Morisada T. (2007) Spreds are essential for embryonic lymphangiogenesis by regulating vascular endothelial growth factor receptor 3 signaling. Mol. Cell. Biol. 27, 4541–4550. 17438136 10.1128/MCB.01600-06 PMC1900061

[73] Denayer E. Ahmed T. Brems H. Van Woerden G. Borgesius N.Z. Callaerts-Vegh Z. (2008) Spred1 is required for synaptic plasticity and hippocampus-dependent learning. J. Neurosci. 28, 14443–14449. 19118178 10.1523/JNEUROSCI.4698-08.2008 PMC6671253

[74] Brems H. Chmara M. Sahbatou M. Denayer E. Taniguchi K. Kato R. (2007) Germline loss-of-function mutations in SPRED1 cause a neurofibromatosis 1-like phenotype. Nat. Genet. 39, 1120–1126. 17704776 10.1038/ng2113

[75] Messiaen L. Yao S. Brems H. Callens T. Sathienkijkanchai A. Denayer E. (2009) Clinical and mutational spectrum of neurofibromatosis type 1-like syndrome. JAMA 302, 2111–2118. 19920235 10.1001/jama.2009.1663

[76] Pasmant E. Gilbert-Dussardier B. Petit A. de Laval B. Luscan A. Gruber A. (2015) SPRED1, a RAS MAPK pathway inhibitor that causes Legius syndrome, is a tumour suppressor downregulated in paediatric acute myeloblastic leukaemia. Oncogene 34, 631–638. 24469042 10.1038/onc.2013.587

[77] Brems H. Legius E. (2013) Legius syndrome, an Update. Molecular pathology of mutations in SPRED1. Keio J. Med. 62, 107–112. 24334617 10.2302/kjm.2013-0002-re

[78] Rauen K.A. (2013) The RASopathies. Annu. Rev. Genomics Hum. Genet. 14, 355–369. 23875798 10.1146/annurev-genom-091212-153523 PMC4115674

[79] Yoshida T. Hisamoto T. Akiba J. Koga H. Nakamura K. Tokunaga Y. (2006) Spreds, inhibitors of the Ras/ERK signal transduction, are dysregulated in human hepatocellular carcinoma and linked to the malignant phenotype of tumors. Oncogene 25, 6056–6066. 16652141 10.1038/sj.onc.1209635

[80] Lorenzo C. McCormick F. (2021) SPRED proteins and their roles in signal transduction, development, and malignancy. Genes Dev. 34, 1410–1421. 10.1101/gad.341222.120 PMC7608746 33872193

[81] Ablain J. Xu M. Rothschild H. Jordan R.C. Mito J.K. Daniels B.H. (2018) Human tumor genomics and zebrafish modeling identify SPRED1 loss as a driver of mucosal melanoma. Science 362, 1055–1060. 30385465 10.1126/science.aau6509 PMC6475924

[82] Ablain J. Liu S. Moriceau G. Lo R.S. Zon L.I. (2021) SPRED1 deletion confers resistance to MAPK inhibition in melanoma. J. Exp. Med. 218, e20201097. 33306107 10.1084/jem.20201097 PMC7927430

[83] Nonami A. Taketomi T. Kimura A. Saeki K. Takaki H. Sanada T. (2005) The Sprouty-related protein, Spred-1, localizes in a lipid raft/caveola and inhibits ERK activation in collaboration with caveolin-1. Genes Cells 10, 887–895. 16115197 10.1111/j.1365-2443.2005.00886.x

[84] Hirata Y. Brems H. Suzuki M. Kanamori M. Okada M. Morita R. (2016) Interaction between a domain of the negative regulator of the Ras-ERK pathway, SPRED1 protein, and the GTPase-activating protein-related domain of neurofibromin is implicated in Legius syndrome and neurofibromatosis type 1. J. Biol. Chem. 291, 3124–3134. 26635368 10.1074/jbc.M115.703710 PMC4751360

[85] Stowe I.B. Mercado E.L. Stowe T.R. Bell E.L. Oses-Prieto J.A. Hernández H. (2012) A shared molecular mechanism underlies the human rasopathies Legius syndrome and Neurofibromatosis-1. Genes Dev. 26, 1421–1426. 22751498 10.1101/gad.190876.112 PMC3403010

[86] Yan W. Markegard E. Dharmaiah S. Urisman A. Drew M. Esposito D. (2020) Structural insights into the SPRED1-neurofibromin-KRAS complex and disruption of SPRED1-neurofibromin interaction by oncogenic EGFR. Cell Rep. 32, 107909. 32697994 10.1016/j.celrep.2020.107909 PMC7437355

[87] Liu Z. Filip I. Gomez K. Engelbrecht D. Meer S. Lalloo P.N. (2020) Genomic characterization of HIV-associated plasmablastic lymphoma identifies pervasive mutations in the JAK-STAT pathway. Blood Cancer Discov. 1, 112–125. 33225311 10.1158/2643-3230.BCD-20-0051 PMC7679070

[88] Melzner I. Bucur A.J. Brüderlein S. Dorsch K. Hasel C. Barth T.F. (2005) Biallelic mutation of SOCS-1 impairs JAK2 degradation and sustains phospho-JAK2 action in the MedB-1 mediastinal lymphoma line. Blood 105, 2535–2542. 15572583 10.1182/blood-2004-09-3701

[89] Schif B. Lennerz J.K. Kohler C.W. Bentink S. Kreuz M. Melzner I. (2013) SOCS1 mutation subtypes predict divergent outcomes in diffuse large B-cell lymphoma (DLBCL) patients. Oncotarget 4, 35–47. 23296022 10.18632/oncotarget.774 PMC3702206

[90] Yoshimura A. Ito M. Chikuma S. Akanuma T. Nakatsukasa H. (2018) Negative regulation of cytokine signaling in immunity. Cold Spring Harb. Perspect. Biol. 10, a028571. 28716890 10.1101/cshperspect.a028571 PMC6028070

[91] Thaventhiran J.E.D. Lango Allen H. Burren O.S. Rae W. Greene D. Staples E. (2020) Whole-genome sequencing of a sporadic primary immunodeficiency cohort. Nature 583, 90–95. 32499645 10.1038/s41586-020-2265-1 PMC7334047

[92] Hadjadj J. Castro C.N. Tusseau M. Stolzenberg M.C. Mazerolles F. Aladjidi N. (2020) Early-onset autoimmunity associated with SOCS1 haploinsufficiency. Nat. Commun. 11, 5341. 33087723 10.1038/s41467-020-18925-4 PMC7578789

[93] Lee P.Y. Platt C.D. Weeks S. Grace R.F. Maher G. Gauthier K. (2020) Immune dysregulation and multisystem inflammatory syndrome in children (MIS-C) in individuals with haploinsufficiency of SOCS1. J. Allergy Clin. Immunol. 146, 1194–1200.e1191. 32853638 10.1016/j.jaci.2020.07.033 PMC7445138

[94] Horino J. Fujimoto M. Terabe F. Serada S. Takahashi T. Soma Y. (2008) Suppressor of cytokine signaling-1 ameliorates dextran sulfate sodium-induced colitis in mice. Int. Immunol. 20, 753–762. 18381351 10.1093/intimm/dxn033

[95] Tanaka K. Ichiyama K. Hashimoto M. Yoshida H. Takimoto T. Takaesu G. (2008) Loss of suppressor of cytokine signaling 1 in helper T cells leads to defective Th17 differentiation by enhancing antagonistic effects of IFN-γ on STAT3 and Smads. J. Immunol. 180, 3746–3756. 18322180 10.4049/jimmunol.180.6.3746

[96] Chikuma S. Kanamori M. Mise-Omata S. Yoshimura A. (2017) Suppressors of cytokine signaling: Potential immune checkpoint molecules for cancer immunotherapy. Cancer Sci. 108, 574–580. 28188673 10.1111/cas.13194 PMC5406529

[97] Lawson K.A. Sousa C.M. Zhang X. Kim E. Akthar R. Caumanns J.J. (2020) Functional genomic landscape of cancer-intrinsic evasion of killing by T cells. Nature 586, 120–126. 32968282 10.1038/s41586-020-2746-2 PMC9014559

[98] Wei J. Long L. Zheng W. Dhungana Y. Lim S.A. Guy C. (2019) Targeting REGNASE-1 programs long-lived effector T cells for cancer therapy. Nature 576, 471–476. 31827283 10.1038/s41586-019-1821-z PMC6937596

[99] Shifrut E. Carnevale J. Tobin V. Roth T.L. Woo J.M. Bui C.T. (2018) Genome-wide CRISPR screens in primary human T cells reveal key regulators of immune function. Cell 175, 1958–1971.e1915. 30449619 10.1016/j.cell.2018.10.024 PMC6689405

[100] Ji Y. Wrzesinski C. Yu Z. Hu J. Gautam S. Hawk N.V. (2015) miR-155 augments CD8^+^ T-cell antitumor activity in lymphoreplete hosts by enhancing responsiveness to homeostatic γ_C_ cytokines. Proc. Natl. Acad. Sci. U.S.A. 112, 476–481. 25548153 10.1073/pnas.1422916112 PMC4299215

[101] Tang B. Wang Z.R. Qi G.Y. Yuan S.G. Yu S.P. Li B. (2015) MicroRNA-155 deficiency attenuates ischemia-reperfusion injury after liver transplantation in mice. Transpl. Int. 28, 751–760. 25611689 10.1111/tri.12528

[102] Takahashi R. Yoshimura A. (2014) SOCS1 and regulation of regulatory T cells plasticity. J. Immunol. Res. 2014, 943149. 25133199 10.1155/2014/943149 PMC4123481

[103] Lu L.F. Thai T.H. Calado D.P. Chaudhry A. Kubo M. Tanaka K. (2009) Foxp3-dependent MicroRNA155 confers competitive fitness to regulatory T cells by targeting SOCS1 protein. Immunity 30, 80–91. 19144316 10.1016/j.immuni.2008.11.010 PMC2654249

[104] Takahashi R. Nishimoto S. Muto G. Sekiya T. Tamiya T. Kimura A. (2011) SOCS1 is essential for regulatory T cell functions by preventing loss of Foxp3 expression as well as IFN-γ and IL-17A production. J. Exp. Med. 208, 2055–2067. 21893603 10.1084/jem.20110428 PMC3182063

[105] Chang J.H. Xiao Y.C. Hu H.B. Jin J. Yu J.Y. Zhou X.F. (2012) Ubc13 maintains the suppressive function of regulatory T cells and prevents their conversion into effector-like T cells. Nat. Immunol. 13, 481–490. 22484734 10.1038/ni.2267 PMC3361639

[106] Takimoto T. Wakabayashi Y. Sekiya T. Inoue N. Morita R. Ichiyama K. (2010) Smad2 and Smad3 are redundantly essential for the TGF-β-mediated regulation of regulatory T plasticity and Th1 development. J. Immunol. 185, 842–855. 20548029 10.4049/jimmunol.0904100

[107] Lu L.F. Boldin M.P. Chaudhry A. Lin L.L. Taganov K.D. Hanada T. (2010) Function of miR-146a in controlling Treg cell-mediated regulation of Th1 responses. Cell 142, 914–929. 20850013 10.1016/j.cell.2010.08.012 PMC3049116

[108] Lu L.F. Gasteiger G. Yu I.S. Chaudhry A. Hsin J.P. Lu Y.H. (2015) A single miRNA-mRNA interaction affects the immune response in a context- and cell-type-specific manner. Immunity 43, 52–64. 26163372 10.1016/j.immuni.2015.04.022 PMC4529747

[109] Sekiya T. Kashiwagi I. Yoshida R. Fukaya T. Morita R. Kimura A. (2013) Nr4a receptors are essential for thymic regulatory T cell development and immune homeostasis. Nat. Immunol. 14, 230–237. 23334790 10.1038/ni.2520

[110] Sekiya T. Kashiwagi I. Inoue N. Morita R. Hori S. Waldmann H. (2011) The nuclear orphan receptor Nr4a2 induces Foxp3 and regulates differentiation of CD4^+^ T cells. Nat. Commun. 2, 269. 21468021 10.1038/ncomms1272 PMC3104557

[111] Sekiya T. Kondo T. Shichita T. Morita R. Ichinose H. Yoshimura A. (2015) Suppression of Th2 and Tfh immune reactions by Nr4a receptors in mature T reg cells. J. Exp. Med. 212, 1623–1640. 26304965 10.1084/jem.20142088 PMC4577835

[112] Martinez G.J. Pereira R.M. Aijo T. Kim E.Y. Marangoni F. Pipkin M.E. (2015) The transcription factor NFAT promotes exhaustion of activated CD8^+^ T cells. Immunity 42, 265–278. 25680272 10.1016/j.immuni.2015.01.006 PMC4346317

[113] Mognol G.P. Spreafico R. Wong V. Scott-Browne J.P. Togher S. Hoffmann A. (2017) Exhaustion-associated regulatory regions in CD8^+^ tumor-infiltrating T cells. Proc. Natl. Acad. Sci. U.S.A. 114, E2776–E2785. 28283662 10.1073/pnas.1620498114 PMC5380094

[114] Chen J. López-Moyado I.F. Seo H. Lio C.J. Hempleman L.J. Sekiya T. (2019) NR4A transcription factors limit CAR T cell function in solid tumours. Nature 567, 530–534. 30814732 10.1038/s41586-019-0985-x PMC6546093

[115] Liu X. Wang Y. Lu H. Li J. Yan X. Xiao M. (2019) Genome-wide analysis identifies NR4A1 as a key mediator of T cell dysfunction. Nature 567, 525–529. 30814730 10.1038/s41586-019-0979-8 PMC6507425

[116] Sekiya T. Kagawa S. Masaki K. Fukunaga K. Yoshimura A. Takaki S. (2021) Regulation of peripheral Th/Treg differentiation and suppression of airway inflammation by Nr4a transcription factors. iScience 24, 102166. 33665581 10.1016/j.isci.2021.102166 PMC7907427

[117] Hibino S. Chikuma S. Kondo T. Ito M. Nakatsukasa H. Omata-Mise S. (2018) Inhibition of Nr4a receptors enhances antitumor immunity by breaking Treg-mediated immune tolerance. Cancer Res. 78, 3027–3040. 29559474 10.1158/0008-5472.CAN-17-3102

